# Designing Natural Rubber Shape Stabilized Phase Change Materials: Impact of Matrix Network on Thermophysical Properties

**DOI:** 10.3390/molecules31020390

**Published:** 2026-01-22

**Authors:** Marc Neira-Viñas, Nicolas Candau, Ana Inés Fernández

**Affiliations:** 1 DIOPMA Research Group, Departament de Ciència de Materials i Química Física, Universitat de Barcelona, 08028 Barcelona, Spain; marc.neira@ub.edu; 2 eb-POLICOM—e-PLASCOM, Departament de Ciència i Enginyeria de Materials, Escola d’Enginyeria de Barcelona-Est (EEBE), Universitat Politècnica de Catalunya, BarcelonaTech (UPC), Av. Eduard Maristany 16, 08019 Barcelona, Spain; nicolas.candau@upc.edu; 3 Barcelona Research Centre in Multiscale Science and Engineering, Universitat Politècnica de Catalunya, BarcelonaTech (UPC), Av. Eduard Maristany, 16, 08019 Barcelona, Spain

**Keywords:** shape-stabilized phase change materials, natural rubber, differential scanning calorimetry, thermal energy storage, crosslinked elastomers, confinement, melting point depression

## Abstract

Shape-stabilized phase change materials (SSPCMs) have been a promising thermal energy storage (TES) solution to combine the high energy density of solid-to-liquid (SL) PCMs and the structural stability of solid–solid PCMs. Although polymeric matrices have been used for their reduced cost and ease of processability, few have evaluated the use of crosslinked natural rubber (NR). In this study, we evaluate by differential scanning calorimetry (DSC) the preparation of room-temperature tailorable SSCPMs by the design of NR matrices with different crosslink density vulcanized by dicumyl peroxide (DCP) or sulphur, with special focus on the quantification of the content of PCM. The results indicate that the amount of PCM stable in the NR matrix is low, with PCM contents between 16 and 24% and enthalpies between 16 and 20 J·g^−1^. Likewise, it is well-known that thermophysical properties of the PCMs vary upon confinement in a small-scale porous matrix. The confinement of the PCM in the rubber network results in a measured enthalpy below the expected value, and a melting point depression of up to 23.6 °C, dependent on crosslink density. These results highlight the structural complexity of NR-PCM composites and the need for further investigation.

## 1. Introduction

The increasing global energy demand and the deployment of sustainable energy sources capacity have made energy storage systems a key player in the energy transition. Thermal energy storage (TES) systems allow to temporarily store heat for later use, from daily, i.e., passive heating and cooling for household ambient conditioning, to seasonal storage such as underground heat storage for district heating. Moreover, they enable the recovery of heat from industrial processes or the decoupling of the supply and demand of energy, like in concentrated solar power plants [1].

One of the main types of TES systems is those based on latent heat storage, in which heat is stored through the phase transition of a phase change material (PCM) and released in the inverse phase transition. These types of systems benefit from the fact that a large amount of heat can be stored in an isothermal process in comparison to sensible heat, where a temperature increase in the material is intrinsic to its functioning principle. However, PCM can present some drawbacks related to the phase transformations that stall its potential applications. Solid-to-liquid (S-L) PCMs are mainly used due to their high latent heat, but are usually compromised by their lack of structure in the liquid state. This requires some form of containment, where problems like leakage or volume changes due to the phase transition may affect heat transfer and overall efficiency. Although employing materials with solid-to-solid (S-S) phase transitions has been a promising solution to overcome these issues, they generally still present lower transition enthalpies than their S-L PCM counterparts.

To address these limitations, shape-stabilized phase change materials (SSPCMs) have emerged as a novel type of heat storage media. By confining an S-L PCM inside a solid matrix capable of retaining the shape upon melting of the PCM, SSPCMs combine both the structural integrity of S-S PCMs and the higher energy density of S-L PCMs without the need for micro or microencapsulation, while preventing leakage.

The strategies to develop SSPCMs and their properties may vary greatly depending on the matrix selected as support. There is a large variety of materials selected for this purpose, depending on the compatibility of the PCM with the matrix, the targeted temperature of use, and the desired properties of the final SSPCMs, such as carbon scaffolds, metal oxides, metal foams, or polymers. Porous inorganic frameworks like expanded graphite [2,3,4] or silicon dioxide [5,6], provide high structural and chemical stability, useful for high temperature TES systems where highly corrosive molten salts are usually one of the preferred PCMs. Metal foams provide great structural integrity and increased effective heat transfer [7] due to their high thermal conductivity, which may balance the inherent low thermal conductivity of most organic phase change materials and aid in the melting/solidification cycles. Polymeric networks can also be considered as porous supports to obtain SSPCMs for low and medium temperatures of operation, with low density, high flexibility, and ease of processability [8].

Polymeric matrix-based SSPCMs are most used with organic PCMs due to their compatibility with the most common organic PCMs, such as paraffins, fatty acids, or esters. Thermoplastic polymers such as polyolefin commodities, namely HDPE [9,10] or PP [11], are usually chosen for their ease of mixing with organic PCMs by several techniques such as melt blending or solvent casting [8]. Thermoplastic elastomers such as block copolymers like SEBS [12], copolyesters [13], and ESEBE [14] have also been used as matrices for PCMs. These materials have the properties of thermoplastic matrix-based SSPCMs combined with a high flexibility due to a network structure comprising hard and soft segments. In the case of thermoset elastomers, their compounding with organic PCMs has been thoroughly considered for non-TES purposes such as the development of shape memory polymers [15,16,17,18,19,20] or to enhance the durability of rubber [21,22]. However, little research has been performed with the scope to develop SSPCMs with an elastomeric matrix despite the high flexibility, high dimensional stability upon thermal cycling, and damping properties of NR.

Most of the studies about SSPCMs with crosslinked elastomeric matrices, such as silicone [23,24] or un-crosslinked natural rubber latex [25,26], used the elastomer as a support material for already micro-encapsulated PCMs. However, the use of these materials as a matrix to confine bulk PCM is limited to silicone [27] or EPDM [28]. To the author’s knowledge, only a few studies have used NR as a support material for bulk non-encapsulated PCMs for TES applications [29,30], despite the numerous benefits of NR, such as its hyperelasticity, fatigue resistance, damping properties, and affordability.

Despite the advantages that SSPCMs offer, some challenges arise from the confinement of an S-L PCM inside matrices with nanometric porosity, such as the unpredictability of its thermophysical properties. An extensive review on the topic has been performed by Gao et al. [31], where the variation in latent heat and temperatures of transition is assessed.

Although the results usually show an overall melting point depression and lowering of the latent heat of the PCM, the reason behind these effects is not always clear. Several explanations are usually given to the same phenomena such as interface interactions that limit the crystallization of the PCM [32], permanent non-freezing layers [5], induced metastable phases by surface-freezing like rotor phases in alkanes [33,34] or size effects like the Gibbs–Thomson effect [5,6], which states that the melting point of a component is depressed with a reduction in its size [35,36]. Based on the latter concept, thermoporosimetry has been used to evaluate the pore-size distribution of a porous material through the analysis of the melting and crystallization point depression of a liquid filling its pores [37]. In vulcanized rubber comprising networks [38,39,40,41,42], thermoporosimetry has been employed by swelling NR with different organic solvents to qualitatively and quantitatively study the structure of the network created through the crosslinking of the polymer chains, analogous to the porosity. This gathers a lot of information regarding the impact of the rubber network on the melting point depression and latent heat decrease in several confined liquids.

In this study, we aim to investigate the potential of SSPCMs developed with a natural rubber matrix through the evaluation of the confined PCM thermophysical properties. We hypothesize that the dependence of the melting point of solvents confined within the network with the mesh size created by the crosslinking may appear as a potential route to design SSPCMs with tailored melting temperatures. To validate this approach, a series of NR networks with different vulcanization systems and crosslink densities have been designed and used as SSPCM matrices. An assessment of the preparation of the PCMs was performed, and the resulting SSPCMs have been analysed by differential scanning calorimetry (DSC) to evaluate their TES potential.

## 2. Results and Discussion

### 2.1. NR-FAE

**Figure 1:**
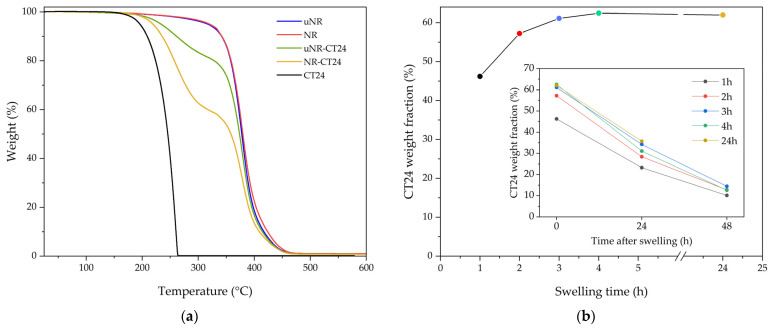
(**a**) TGA analysis of CT24, NR, and the prepared vulcanized and vulcanized SSCPMs. (**b**) CT24 intake during swelling of D1.5 samples. The detailed image shows the mass loss of CT24 during the first 24 h.

**Figure 2:**
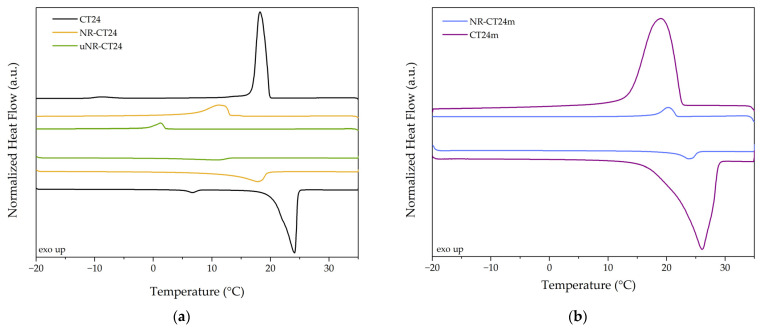
Differential scanning calorimetry plots of (**a**) CT24 and swelling and compounding prepared SSCPCMs and (**b**) CT24m and the SSCPMs prepared by compounding with CT24m.

The difference in the composition of the resulting SSPCMs and the thermophysical properties between the compounding and the swelling approaches to the preparation of NR-CT24 composites can be seen in Table 1 and Figure 1 and Figure 2, respectively. For the compounding method where unvulcanized SSPCMs are prepared (uNR-CT24), the final compound has 9.9 wt.% of PCM, measured by TGA (Figure 1a). From the DSC thermograms presented in Figure 2a, uNR-CT24 melting onset (T_m,o_) and crystallization onset (T_c,o_) are significantly shifted to lower temperatures, from 20.9 °C to −0.1 °C and from 19.9 to 2.1 °C, respectively, and the melting enthalpy is measured to be 13.1 J·g^−1^. Although the lower crystallization temperature of the FAE could be explained by a higher degree of super cooling when CT24 is inside the elastomeric matrix, the lower temperature of melting can be attributed to several reasons: (i) degradation of CT24 during the preparation, since, during mastication at 80 °C, the temperature can rise a few tenths of degrees due to molecular friction and friction of the material with the wall of the mixer, and TGA analysis (Figure 1a) shows that CT24 decomposition starts at 120 °C, or, (ii) confinement effects due to the confinement of CT24 in the rubber matrix.

To discard the possible degradation during compounding, samples of NR crosslinked with 1.5 phr DCP were swollen with CT24 (NR-CT24) after its mastication and vulcanization. Figure 1b shows that equilibrium swelling is reached after 4 h, with 61.3 wt.% CT24. However, blooming of the excess CT24 starts shortly after removing the samples from the liquid CT24. The weight loss of CT24 during the posterior 48 h can be seen in Figure 1b in detail and seems to indicate that all samples tend to approach a similar concentration of equilibrium 12–16 wt.%, similar to what was found by Volynski et al. in vulcanized NR-octadecane systems [43].

The absence of blooming in the compounded samples could be attributed to a higher solubility or lower kinetics of blooming of CT24 in non-vulcanized rubber in comparison to vulcanized rubber. The TGA of a crosslinked sample swollen for 24 h, after 24 h of blooming (Figure 1b), shows a CT24 content of 35.7 wt.%. Its thermophysical properties are measured in Figure 2a. NR-CT24 has a melting enthalpy of 60.0 J·g^−1^, T_m,o_ is reduced by 12.2 °C, and T_c,o_ by 12.6 °C in comparison to bulk CT24 transition temperatures. Since swelling is performed at 40 °C, degradation is unlikely to occur; the variation in the transition temperatures could be related to an interaction between CT24 and the NR matrix.

To confirm this hypothesis, results were compared with samples of NR compounded with microencapsulated CT24m. In Figure 2b, it can be seen that samples with microencapsulated PCM maintain the same transition temperatures as the bulk CT24m, even though they are exposed to the same temperatures as NR-CT24.

However, by observing the DSC curves of CT24, some difficulties can be anticipated when trying to understand the effect of the confinement of the PCM inside the elastomeric matrix on the thermophysical properties of the NR-PCM composites. From the presence of a broad shoulder on the bulk CT24 melting peak and an additional peak at lower temperatures, we can infer that CT24 could be a non-eutectic mixture of two PCMs. Since different chain-length molecules can present different swelling behaviours [15], the lower transition temperature could be partially attributed to a different composition of the PCM inside the matrix, closer to the eutectic point.

To avoid this phenomenon, capric acid (CA) was selected as a PCM. The choice was based on several factors. CA is commonly used as a phase change material [13], is present in the composition of natural rubber [44,45,46], has been used in natural rubber shape memory polymers before [15], and has a melting point (31.6 °C), slightly higher to CT24. NR-CA compounds were prepared solely by swelling since CA degradation temperature is around 80 °C, lower than the mastication temperature of the NR (95 °C) and its vulcanization temperature (170 °C).

### 2.2. NR-CA

**Figure 3:**
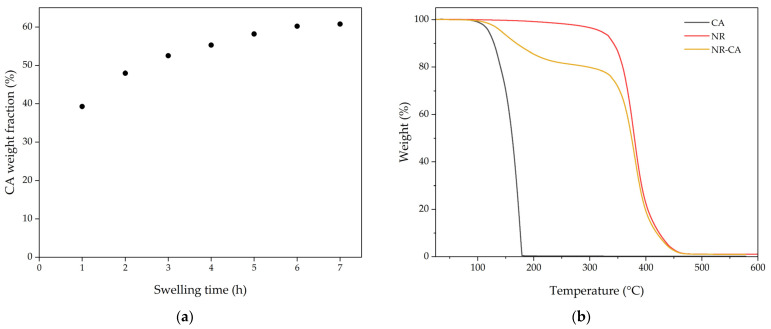
(**a**) CA intake during swelling for D1.5. (**b**) Thermogravimetric analysis of CA, NR, and the NR-CA.

The results of the swelling process of CA in DCP 1.5 phr vulcanized NR can be seen in Figure 3. Although a rapid intake of CA is observed during the first hour, it shows a plateau around 6 h of swelling, reaching a concentration of CA of 60.2%, similar to the values reached using CT24 and to those observed by Pantoja et al. for other fatty acids in crosslinked NR bands [15]. After the swelling, all samples presented blooming of CA on the NR surface. Samples swollen for 6 h were measured by TGA once the blooming stopped, and concentrations of CA in the NR matrix were found to be 18.1%.

**Figure 4:**
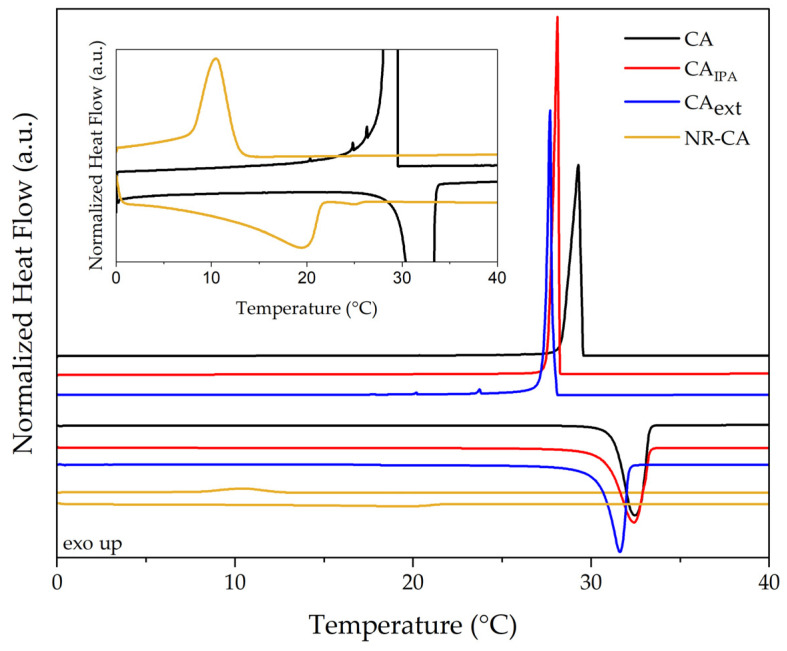
DSC heating and cooling curves of NR-CA SSCPM, CA, and CA extracted with IPA from the NR matrix. In detail, NR-CA and CA close-up for clarity of comparison.

The results of the comparative DSC analysis between bulk CA and NR-CA samples are presented in Figure 4 and Table 2. Capric acid melts and crystallizes in a narrow temperature range with an onset (T_m,o_) and peak (T_m,p_) melting temperature of 31.6 °C and 32.4 °C, respectively, with a melting enthalpy of 155.0 J·g^−1^. However, the NR-CA sample shows a significantly different melting behaviour, with a melting onset and peak of 11.6 °C and 19.9 °C, and broadening of the melting peak. The enthalpy of the NR-CA composite was measured to be 19.7 J·g^−1^.

To rule out a possible CA degradation by a parallel reaction with unreacted peroxide from the vulcanization step, as an explanation for the melting point depression observed in the DSC, the CA embedded in the NR matrix was extracted with 2-propanol (IPA) by immersion for 24 h and crystallised by evaporating IPA at 23 °C in a fume hood. The results can be seen in Figure 4. It can be seen that the CA that melted between 11.6 °C and 25 °C inside the matrix presents a T_m,o_ of 30.5 °C once extracted from the matrix (CA_ext_) similar to that of CA crystalised from IPA (CA_IPA_), which is 30.8 °C, indicating that the melting point depression of the confined CA is not a consequence of a possible degradation. However, its enthalpy is reduced from 148.2 J·g^−1^ to 126.3 J·g^−1^ when comparing the capric acid crystallised from IPA with CA extracted from the rubber matrix. This could be attributed to the presence of components of rubber extracted along with the CA that do not participate in the melting, thus reducing the normalized enthalpy of melting measured.

To evaluate the possibility of obtaining NR-PCM composites with tailored phase change temperatures, samples with different vulcanization systems, such as DCP and sulphur, and different vulcanization agent (VA) concentrations were prepared.

The swelling measurements in cyclohexane presented in Table 3 show that the crosslink density increases linearly with the increasing VA phr content for DCP crosslinked samples, ranging from 1.18·10^−4^ to 2.06·10^−4^ mol·cm^−3^, whereas their sol content decreased monotonically with the lowest sol fraction being 3.0% for the highest crosslinked sample with 2 phr DCP. For sulphur crosslinked samples, the crosslink density increases from 1.35·10^−4^ to 1.66·10^−4^ mol·cm^−3^ for 1 and 1.5 phr S, but is the lowest value is measured samples with 2 phr S (1.05·10^−4^ mol·cm^−3^), probably because of the heterogeneity of the mixture due to a higher phr of S and CBS. Excluding the sample crosslinked with 2 phr S, the measured crosslink densities with 1 phr S and 1.5 phr S agree with those measured previously by Candau et al. [47], where values of 0.99·10^−4^, 1.42·10^−4^, and 1.76·10^−4^ mol·cm^−3^ were reported for NR samples crosslinked with 0.8, 1.2, and 1.6 phr of S. Ikeda et al. [48] reported values of 0.76·10^−4^, 1.38·10^−4^, and 1.32·10^−4^ for samples vulcanized with 1 and 2 phr DCP and 1.5 phr S, respectively. Although these values are lower than the ones presented in this work, this difference could be attributed to the lower temperature of vulcanization used by Ikaeda et al.

Although there was no significant difference in the soluble fraction for samples crosslinked with 1 and 1.5 phr S, there is a clear increase for samples crosslinked with 2 phr S, aligned with the relationship found for DCP crosslinked samples, where lower crosslink density implied higher soluble fraction. It is important to note that the soluble fraction on DCP takes into account only naturally present components in natural rubber, such as proteins or fatty acids, as well as free small molecular weight polymer chains. However, for sulphur crosslinked samples, the additional mass loss of the additives present in the vulcanization system may also contribute to the overall higher soluble fraction.

**Figure 5:**
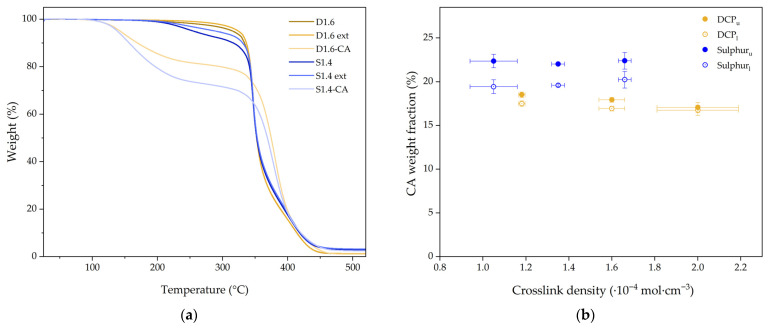
(**a**) TGA of NR before and after the extraction and the corresponding SSPCM for D1.6 and S1.4 samples. (**b**) Lower and upper bounds of the calculated PCM content measured on NR-CA samples.

The vulcanized NR samples were swollen in 15 mL CA for 6 h. After extracting the samples, blooming was observed readily in the form of crystallized CA on the NR surface due to the lowering of the solubility with the decrease in temperature [15]. Blooming kinetics were also found to be largely influenced by the crosslinked density of the samples, as observed by Volynski [43]. Samples with a higher crosslink density, such as S1.7 and D2, did not present blooming surface CA after the second removal (48 h) of excess CA crystallized in the NR surface, while samples with lower crosslink density required over 120 h for the blooming to stop. As can be seen in Figure 5a, quantification of the real content of CA inside the samples by TGA is not direct. Natural rubber samples present significant weight loss at the end of the CA weight loss step in NR-CA samples (270 °C), especially in the case of sulphur crosslinked samples. Moreover, there is a difference in the mass loss at 270 °C for as-prepared samples and samples after swelling with cyclohexane, probably due to the extraction of low molecular weight components during the swelling in the rubber. Since there is no method to know how much soluble compounds are really extracted during the CA swelling, upper and lower bounds of PCM content have been defined. The lower bound considers that no soluble compounds are extracted, and the upper bound considers that CA is capable of extracting the same number of components as cyclohexane. Moreover, for samples with different crosslink densities, mass loss at the considered temperature is different; thus, every NR-CA sample must be corrected according to Equations (1) and (2):

(1) $$w_{CA,u}=w_{T}(270\;{}^{\circ}C)-w_{NR}(270\;{}^{\circ}C)$$

(2) $$w_{CA,\;l}=w_{T}\left(270\;{}^{\circ}C\right)-w_{NR,\;ext}(270\;{}^{\circ}C)$$

where *w_CA,u_* [-] and *w_CA,l_* [-] are the upper and lower bounds of calculated CA mass fraction in the SSCPCM, *w_T_* (270 °C) [-] is the total mass fraction of the SSPCM loss at 270 °C, and *w_NR_* [-] and w*_NR,ext_* [-] are the mass fraction loss measured at 270 °C of NR and NR after the crosslink density measurement, respectively.

The calculated upper and lower amounts of CA upon stabilization can be seen in Figure 5b and Table 4. The results presented in the table show that the difference between upper and lower CA weight fraction approximations is the lowest for higher crosslinked samples of each vulcanization system D2 and S1.7 Moreover, D1.2 and D1.6 and S1.4 and S1.7 present similar differences between upper and lower approximations, but D2 and S1.1 deviate more clearly, which is in good agreement with the values reported from the soluble fraction in the crosslink density measurements (Table 3).

From Figure 5b, it can be seen that the CA intake on the SSCPM is slightly inversely dependent on the crosslink density of the material for DCP samples, similarly to what would be expected from saturated swollen samples. However, no significant dependence was found for sulphur crosslinked samples. This could be attributed to the slow kinetics of blooming, which may take hundreds of days to reach stability [43], or to the additional additives inside the rubber matrix, whose concentration in the NR and degradation kinetics are not known precisely, thus affecting the calculated ratio of CA.

**Figure 6:**
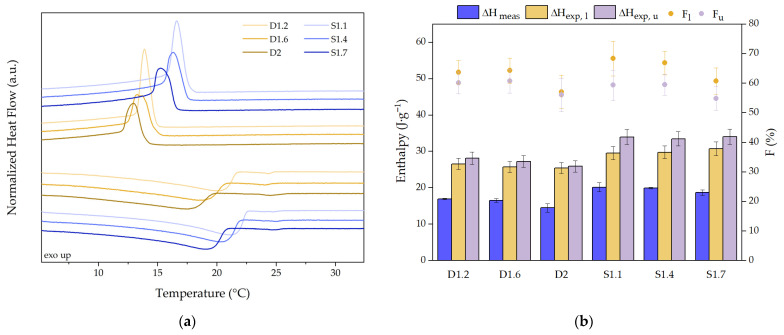
(**a**) DSC heat flow curves of the prepared NR-CA SSPCMs. (**b**) Enthalpy measured, expected enthalpy, and F for the lower and upper bounds of PCM.

The DSC curves of the prepared SSPCMs are presented in Figure 6a. Figure 6b presents the enthalpy of melting (ΔH_m_) of the NR-CA prepared composites. The measured enthalpies are in the range of 14.5 J·g^−1^ to 16.9 J·g^−1^ for DCP crosslinked samples, and 18.7 J·g^−1^ to 20.2 J·g^−1^ for sulphur crosslinked samples. The results also show that DCP crosslinked samples, the sample with higher crosslink density, D2, presents the lowest melting enthalpy, while no significant difference is found for sulphur crosslinked samples, which agrees with the CA intake values observed in Figure 5b.

Moreover, the melting enthalpy for the prepared SSCPMs is below the expected one for the calculated amount of present CA inside the matrix, independently of considering the upper or lower limit of CA content. This can usually be represented with the weight fraction of crystalline phase F presented in Equation (3):

(3) $$F(\%)=\frac{∆H_{SSPCM}}{∆H_{PCM}\cdot w}\cdot 100$$

F [-] is the ratio of measured enthalpy of the SSPCMs (*∆H_SSPCM_* [J·g^−1^]) and the bulk PCM melting enthalpy (*∆H_PCM_* [J·g^−1^]), measured as 155.0 J·g^−1^ for CA, multiplied by the weight fraction of PCM in the NR (*w* [-]), that will vary depending on the PCM intake limit considered.

For DCP crosslinked samples, F values are between 56% and 61% for the higher limit and between 57% and 64% for the lower limit. For sulphur crosslinked samples, F values are between 55% and 59% for the higher limit and from 61% to 68% for the lower limit. In both systems, the highest crosslinked sample presents the lowest value, which can be attributed to the dependence of the melting enthalpy on the size of the confining space [49]. The lower measured enthalpy of confined liquids in comparison with the enthalpy of the bulk PCM is a phenomenon that has been reported extensively but has been associated with different reasons as the reduction in enthalpy of melting with the crystallite size [50], melting temperature [51] or the presence of a non-freezing part of the PCM [5,36,49] that does not contribute to the enthalpy measured. For CA in confined spaces, the latter explanation has been given for silica-FA composites [6,49]. For solvents confined in natural rubber networks, the enthalpy should decrease with a decrease in the melting temperature due to the presence non-freezing solvent dissolved in the gel and the interaction of solvent molecules with the polymer [42], similar to what was proposed by Volynski et al., who also reported reduced enthalpy of melting for NR-octadecane mixtures, both crosslinked [43] and uncrosslinked [50].

**Figure 7:**
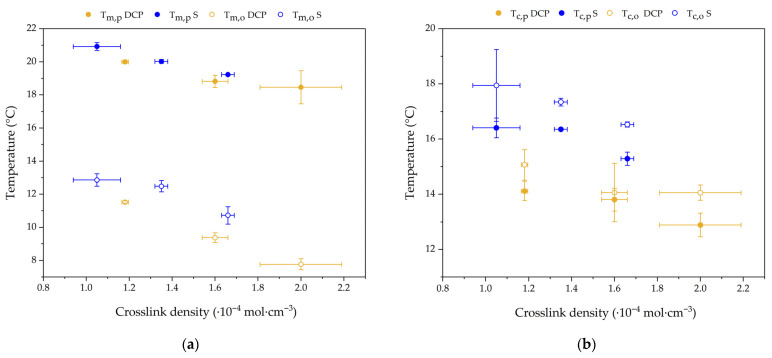
(**a**) Melting and (**b**) crystallization onset and peak transition temperatures of NR-CA SSPCMs against crosslink density.

The characteristic temperatures of melting and crystallization of NR-CA compounds are presented in Figure 7. It is shown that the phase transition of CA confined in the NR matrix is shifted towards lower temperatures in comparison to bulk CA, which, from Figure 4, has a melting onset and peak of 31.6 °C and 32.1 °C and a crystallization onset and peak of 28.3 °C and 29.4 °C. All characteristic transition temperatures seem to decrease with the increasing crosslinking density for both vulcanization systems, and the melting point depression is more pronounced in DCP crosslinked samples. The maximum melting point depression (ΔT_m_) is obtained for D2, which has a melting onset of 7.5 °C and a melting peak of 18.5 °C, or a melting point depression of the melting onset (ΔT_m,o_) and peak (ΔT_m,p_) of 24.1 °C and 14.1 °C, respectively. For samples crosslinked with sulphur, higher overall melting and crystallization temperatures are observed, with the lowest melting onset and peak temperatures measured in S1.7 being 10.6 °C and 19.5 °C, respectively, which means a ΔT_m,o_ and ΔT_m,p_ of 21 and 12.6 °C.

Although T_o_ and T_p_ might serve as a guideline to compare the melting point depression to the crosslink density of the samples, they do not cover the full extent of the phenomenon. Even though T_o_ is usually presented as the melting temperature of a pure material, the broadening of the melting peaks in the DSC thermogram, as seen in Figure 6a, results in the difficulty of measuring a precise T_o_ and, due to the shape of the melting peak, the real magnitude of the melting point depression seems to be underestimated by this metric. This broadening of the peak of melting might be interpreted as the presence of several pore sizes associated with a distribution of crosslink density, but also the heterogeneity of the rubber network. The existence of different domains with different crosslink densities could create a distribution of crystallite sizes, resulting in the distribution of the melting over a broader range [41,47], as crosslinks are expected to be expelled from the crystallites. An exhaustive analysis of the thermogram morphology of confined solvents in polymers was performed by Wu and McKenna for PDMS swollen with cyclohexane and cyclooctane [52], where the broadening of the peak was associated with solvent melting at different temperatures due to the existence of solvent dissolved in the gel and solvent on the surface of the gel. However, this is not expected in the NR-CA studied since the absence of a melting peak at the CA bulk temperature indicates that there is no unconfined CA in the NR-CA measured DSC samples.

For organic solvents confined in a polymer network, the melting point depression is also dependent on the volumetric concentration [38,50,52,53,54] and can be explained by the polymer-solvent interaction defined by the Flory–Huggins solution theory, which states that the melting point of a solvent in solution with a polymer is reduced:

(4) $$\frac{1}{T_{m_{s}}}-\frac{1}{T_{m_{s}^{0}}}=\frac{R}{∆H_{m_{s}}}(\ln\left(1-\phi_{r}\right)+\phi_{r}+\chi \phi_{r}^{2})$$

where T_m,s_ and T_m,s_^o^ are the melting temperature [K] of the bulk and confined solvent, R [J·K^−1^·mol^−1^] is the ideal gas constant, ΔH_m,s_ [J·mol^−1^] is the melting enthalpy of the solvent, ϕ_r_ [-] is the volumetric fraction of rubber, and ꭕ [-] is the Flory–Huggins polymer–solvent interaction parameter.

**Figure 8:**
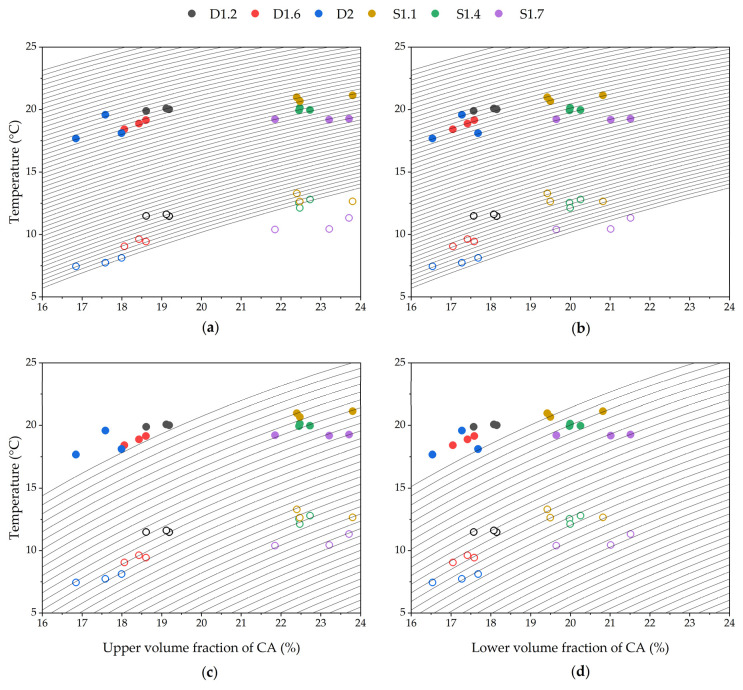
Temperature peak (full circles) and onset of melting (empty circles) of the NR-CA SSCPMs against calculated upper (**a**,**c**) and lower (**b**,**d**) volume fraction of PCM. In the background Flory–Huggins equation is plotted for χ every 0.02(-) between 0 (lower T) and 1 (higher T) and considering: (**a,b**) bulk enthalpy of CA 155 J·g^−1^ and (**c**,**d**) reduced enthalpy of 77.5 J·g^−1^ (F = 0.5).

Since Flory–Huggins is only valid for melting processes and not crystallization, to evaluate the effect of the crosslink density and the volumetric concentration of CA to NR, the T_m,o_ and T_m,p_ results of the prepared NR-CA SSPCMs have been plotted against volume concentration of CA in Figure 8. For DCP crosslinked samples, since only CA and rubber are in the composite, the volume fraction of CA was calculated as:

(5) $$\phi_{CA}=\frac{\frac{m_{CA}}{\rho_{CA}}}{\frac{m_{r}}{\rho_{r}}+\frac{m_{CA}}{\rho_{CA}}}$$

where *m_CA_* and *m_r_* are the mass of CA and rubber in the sample, and *ρ_CA_* and *ρ_r_* are the densities of CA and NR taken as 0.910 and 0.893 g·cm^−3^, respectively. For sulphur crosslinked samples, the volume was corrected for the amount of ZnO present in each sample according to Equation (10), as is explained in the crosslink measurements method section; thus,

(6) $$\phi_{CA}=1-\phi_{r}^{\prime }$$

where $\phi_{r}^{\prime }$ is the corrected volume fraction of rubber in the sample.

The Flory–Huggins interaction parameter (χ) has not been reported in the literature for NR-CA or NR-FA, but was measured as a fitting parameter in a non-linear least squares fit for several fatty acids by Pantoja et al. [15], giving values between 0.73 and 0.65 for lauric, myristic, stearic, and palmitic acid. Moreover, the use of the bulk or confined melting enthalpy in Equation (8) has also been a matter of discussion [41]. Volynski et al. proposed that the reduced enthalpy must be used since crystallite size and non-freezing solvent must be considered [50]. In Figure 8, the FH equation is plotted for NR-CA with interaction parameters between 0 and 1 in the background as a guideline to estimate the effect of concentration on the onset and peak temperature of melting, considering both the upper (Figure 8a,c) and lower (Figure 8b,d) calculated content of CA. In Figure 8a,b FH equation is plotted with the measured bulk enthalpy for capric acid, while in Figure 8c,d, FH is plotted with the calculated melting enthalpy of confined CA for an extreme condition of F = 0.5.

The results show that for sulphur samples, there is no influence of the melting peak or onset temperatures with the concentration of PCM within samples with the same crosslink density.

When evaluating onset temperatures of melting for DCP crosslinked samples, it does not seem that the volume fraction of CA within the same crosslink density samples affects T_m,o_ for D1.2 or D1.6 samples, but it does slightly for D2. In the case of T_m,p_, D1.6 seems to have a dependence on the volumetric concentration but not D2 or D1.2. The combining effect of crosslinking and volume fraction has been studied by Qin and McKenna for NR-hexadecane systems [41], and tried to be formally combined in a single equation able to predict the melting point depression of a solvent confined in polymer networks, with an additional third contribution from the elastic force of the matrix [53] or considering trapped entanglements [54]. Although this approach is outside of the scope of this paper, the narrow range of NR-CA mixtures prepared does not allow for evaluating the contribution of the CA load to the melting point depression of the composite. Mixtures with a broader range of CA fractions should be intentionally prepared with the aim of evaluating each contribution on the melting point precisely.

## 3. Materials and Methods

### 3.1. Materials

The natural rubber (NR) used in this study is SMR (Standard Malaysian Rubber) CV60 (Mooney Viscosity ML 1 + 4, 100 °C: 55–60), supplied by the company Akrochem (Akron, OH, USA), with 0.15% of hydroxylamine added to the latex stage to prevent the raw rubber from stiffening while storing. Sulphur powder S −325 mesh, 99.5% purity, stearic acid (SA) of 97% purity, and zinc oxide (ACS reagent, 99%) were provided by ThermoScientific Chemicals (Waltham, MA, USA). *N*-Cyclohexyl-2-benzothiazolylsulfenamide (CBS) of 98% purity provided by TCI America (Portland, OR, USA). N1-(4-Methylpentan-2-yl)-N4-phenylbenzene-1,4-diamine (6PPD) of 98% purity was purchased from CYMIT Quimica SL (Barcelona, Spain). Cyclohexane 99.5% was purchased from PanReac Applichem ITW Reagents (Darmstadt, Germany).

The PCMs used in this study were bulk CRODA 24 LQ-(GD) (CT24) and CRODA ME 24 XD (CT24m), a slurry with ~60 wt. % solid content of microencapsulated PCM, bio-based fatty acid ester (FAE)-based PCMs provided by Croda Ibérica SAU (Barcelona, Spain)and Capric acid (CA) of 98% purity, purchased from Merck Life Sciences (Darmstadt, Germany).

### 3.2. Sample Preparation

#### 3.2.1. Unvulcanized Samples

The NR was masticated in batches of a total content of 40 g inside the chamber of an internal mixer, Brabender Plastic-Corder W50EHT (Brabender GmbH and Co., Duisburg, Germany), with a chamber volume of 55 cm^3^ at a temperature of 80 °C and a rotation speed of 40 rpm. For the unvulcanized NR-PCM samples, rubber was initially masticated for 5 min, and then 20 wt.% of the corresponding PCM was incorporated and mixed for 10 min. CT24 was incorporated in liquid form, whereas CT24m was incorporated in a slurry as-received (60 wt.% in solid content).

#### 3.2.2. Vulcanized Samples

For samples crosslinked using DCP as vulcanizing agent, NR was masticated for 5 min. Then, the vulcanizing agent dicumyl peroxide (DCP) was added (1, 1.5, or 2 parts per hundred rubber (phr) of the NR) and mixed for 10 additional minutes. For the sulphur crosslinked samples, after 5 min of mastication, antioxidant N-(1,3-dimethylbutyl)-N′-phenyl-p-phenylenediamine (6PPD), stearic acid (SA), and zinc oxide (ZnO) were added. After 2 more minutes, sulphur (S), together with the accelerator N-cyclohexyl-2-benzothiazolesulfenamide (CBS), was added, and the batch was mixed until reaching a total time of 15 min.

The master batch was vulcanized under an IQAP LAP PL-15 hot plate press (IQAP Masterbatch SL, Barcelona, Spain) at 170 °C under 4 MPa for an estimated optimal time for each composition. The result was NR sheets with a thickness of 0.5 mm. The composition of the vulcanized samples can be found in Table 5.

To prepare vulcanized rubber with PCM samples (NR-PCM), 10 × 10 × 0.5 mm NR samples were weighed and immersed in 15 mL of the corresponding PCM, either CT24 or capric acid, for several swelling times. After the swelling was performed, the excess PCM from the surface was removed, and the sample was weighed. Samples were stored in the dark at room temperature (21–23 °C), and their surfaces were cleaned by removing the excess PCM daily until no excess PCM was observed at the surface. The fraction of PCM in the composites (*w_PCM_* [-]) was measured with Equation (7) as the difference in weight:

(7) $$w_{PCM}=\frac{m_{i}-m_{f}}{m_{i}}$$

where *m_i_* [g] is the initial mass of the rubber sample and *m_f_* [g] is the mass at the time of evaluation of the PCM content.

### 3.3. Characterization

#### 3.3.1. Differential Scanning Calorimetry (DSC)

Differential calorimetry analysis was performed using DSC 3+ from Mettler Toledo (Columbus, OH, USA). Measurements were performed with samples of ~6 mg, using aluminium crucibles sealed with a perforated lid, under a N_2_ flow rate of 50 mL·min^−1^. The heating and cooling rates employed were 2 K·min^−1^ unless otherwise stated for all the tests except in the evaluation of NR-CA samples with different crosslink densities, where 0.5 K·min^−1^ was used to ensure an accurate measure of the melting point. All measurements reported in this work are performed by first heating the sample above its melting point and then cooling and heating the sample at the desired temperature rate; thus, the results presented are the first cooling and the second heating runs.

#### 3.3.2. Thermogravimetric Analysis (TGA)

To study the thermal stability of the starting materials, thermogravimetric analysis of the samples was performed using a TGA550 from TA Instruments (New Castle, DE, USA). Measurements were performed from 25 to 600 °C at 10 K·min^−1^ in Pt pans under 90 mL·min^−1^ N_2_ flow rate for the quantification of PCM in the prepared SSPCMs and for the evaluation of the degradation temperatures. To quantify the PCM in the prepared SSPCMs, the same samples used to previously perform the DSC measurements were used. Since non-negligible mass loss from natural rubber can be in the range of the PCM degradation step, the weight fraction of PCM (*w_PCM_* [-]) is calculated taking this into account with the following equation:

(8) $$w_{PCM}=w_{loss}(T_{f,PCM})-w_{loss,\;NR}\;\left(T_{f,PCM}\right)$$

where *w_loss_(T_f,PCM_)* [-] is the total mass fraction lost at the end of the degradation step of the PCM and *w_loss,NR_(T_f,PCM_*) [-] is the mass loss of natural rubber at the same temperature.

#### 3.3.3. Crosslink Density

To measure the average crosslink density of natural rubber, three samples of 10 × 10 × 0.5 mm for each vulcanized rubber composition were cut and weighed (*m_i_* [g]). Samples were then immersed in 15 mL of cyclohexane for 72 h, renewing the solvent every 24 h. The samples were extracted, their surface gently dried with filter paper, and the swollen rubber mass was recorded (*m_sw_* [g]). Then, the swollen samples were dried for 72 h in a fume hood until constant weight, confirmed by vacuum drying, and the dry rubber mass was recorded (*m_d_*). The average network chain density (ν [mol·cm^−3^]) can then be measured from the Flory–Rehner theory with Equation (9) [55]:

(9) $$\nu =\frac{\ln\left(1-\phi_{r}\right)+\phi_{r}+\chi \phi_{r}^{2}}{V(-\phi_{r}^{-\frac{1}{3}}+\frac{2}{f}\phi_{r})}$$

where *V* = 108 [cm^3^·mol^−1^] is the molar volume of cyclohexane and *χ* is the Flory–Huggins polymer–solvent interaction parameter. *χ* = 0.363 [-] was taken for NR swollen with cyclohexane [56]. The crosslink functionality (*f* [-]) was chosen to be equal to 4 [56,57,58]. For DCP vulcanized samples., *ϕ_r_* [-] is the volume fraction of rubber, which is calculated from the dry total mass after the swelling and drying (m_d_ [g]) and the solvent weight (m_sol_ [g]) in the swollen rubber taking 0.910 g·cm^−3^ and 0.777 g·cm^−3^ as the density for rubber and cyclohexane, respectively. Three samples were evaluated for each composition. For sulphur crosslinked samples, the volume fraction of rubber must be corrected to account for the presence of insoluble particles, such as ZnO, and the formation of vacuoles due to these particles [55]:

(10) $$\phi_{r}^{\prime }=\frac{1+\frac{V_{ZnO}}{V_{r}}}{\frac{1}{\phi_{r}^{*}}+\frac{V_{ZnO}}{V_{r}}}=\frac{1+\frac{\frac{x_{ZnO}m_{d}}{\rho_{ZnO}}}{\frac{m_{d}-x_{ZnO}m_{d}}{\rho_{r}}}}{\frac{\frac{m_{d}-x_{ZnO}m_{d}}{\rho_{r}}+\frac{m_{sol}}{\rho_{sol}}}{\frac{m_{d}-x_{ZnO}m_{d}}{\rho_{r}}}+\frac{\frac{x_{ZnO}m_{d}}{\rho_{ZnO}}}{\frac{m_{d}-x_{ZnO}m_{d}}{\rho_{r}}}}$$

where *x_ZnO_* [-] and *ρ*_ZnO_ [g·cm^−3^] are the weight fraction and density (5.61 g·cm^−3^) of ZnO in the dry sample, respectively. This approach assumes CBS, 6PPD, and SA to be completely soluble in cyclohexane. The soluble fraction (*s* [-]) of the samples was measured by the mass loss of soluble components before and after the swelling, according to the equation:

(11) $$s=\frac{m_{i}-m_{d}}{m_{i}}$$

## 4. Conclusions

The potential to use natural rubber as a matrix of SSPCMs with tailorable phase transition temperatures has been evaluated. The study of room-temperature SSPCMs has been limited to single-component PCMs prepared by swelling due to the complexity of the swelling approach and the low degradation temperature of PCMs with solid-to-liquid phase transition around room temperature.

Degradation of the PCM has been discarded as a major contribution to the melting point depression shown by the SSPCM. Crosslink density has been proven to significantly impact the melting point of the SSPCMs, with a higher effect in CA confined in DCP crosslinked NR than in sulphur crosslinked NR. No dependence of the melting point on the volumetric concentration of PCM was observed, probably due to the narrow range of PCM fraction measured in the samples and the difficulty of measuring true values of PCM concentration. The measured enthalpies of melting for sulphur crosslinked samples were overall higher than for DCP crosslinked SSPCMs, due to the higher intake of PCM that was measured by TGA. The enthalpy of melting was lower than the expected enthalpy for all SSPCMs, which could be attributed to a stable non-freezing layer of PCM dissolved in the gel, as proposed in the literature. No significant dependence was found on the crosslink density of the NR matrix, although the highly crosslinked samples of both vulcanization systems showed lower *F* values on average than their less crosslinked counterparts. To measure the contribution of size effects in the measured thermophysical properties, further characterization with X-ray diffraction should be performed to assess the crystallization behaviour of the confined CA, with special focus on the size of the crystal formed upon confinement.

Despite the too low measured enthalpies of melting for the developed SSPCMs to be used for thermal energy storage purposes, the turnability of the transition temperatures shown with modifications in the network structure observed might serve as a baseline to assess the preparation of shape memory polymers and SSPCMs with polymeric networks for temperature ranges where a lack of usable PCMs exists. Moreover, other formulations of NR-PCM should be prepared to assess the dependence of the PCM fraction on the melting point depression and the possibility to increase the amount of PCM stable in the NR matrix.

## Figures and Tables

**Table 1:** Thermophysical properties of CT24 and composites of NR-CT24.

Sample	PCM Weight Fraction (%)	Enthalpy (J·g^−1^)	Melting Onset (°C)	Crystallization Onset (°C)
CT24	100	167.7	20.9	19.9
uNR-CT24	9.9	13.1	−0.1	2.1
NR-CT24	35.7	60.0	8.7	7.3

**Table 2:** Thermophysical properties of CA and NR-CA.

Sample	Enthalpy (J·g^−1^)	Melting Onset (°C)	Melting Peak (°C)
CA	155.0	31.6	32.4
NR-CA	19.7	11.6	19.9
CA_IPA_	148.2	30.5	32.4
CA_ext_	126.3	30.8	31.6

**Table 3:** Cross-link density measurements from the swelling test.

Sample	Vulcanizing Agent (phr)	Crosslink Density (10^4^ mol·cm^−3^)	Soluble Fraction (wt. %)
D1.2	1	1.18 ± 0.02	4 ± 0.2
D1.6	1.5	1.60 ± 0.06	3.9 ± 0.2
D2	2	2.0 ± 0.2	3.0 ± 0.3
S1.4	1	1.35 ± 0.03	5.74 ± 0.7
S1.7	1.5	1.66 ± 0.03	5.22 ± 0.06
S1.1	2	1.05 ± 0.11	7.45 ± 0.11

**Table 4:** Weight loss of SSCPCMs and pristine rubber measured by TGA.

Sample	*w_T_* (270 °C)	*w_NR_* (270 °C)	*w_NR,ext_* (270 °C)	*w_CA,l_*	*w_CA,u_*	*w_CA,l_-w_CA,u_*
D1.2	19.8	2.3	1.3	17.5	18.5	1
D1.6	19.2	2.3	1.3	16.9	17.9	1
D2	18.5	1.8	1.5	16.7	17.1	0.4
S1.1	26.0	6.6	3.6	19.4	22.4	3
S1.4	25.7	6.1	3.7	19.6	22.0	2.4
S1.7	26.4	6.3	4.1	20.2	22.4	2.2

Note: All values are in wt.% and are the mean of 3 measured samples. Standard deviation is not written for clarity but is presented in Figure 6b error bars.

**Table 5:** Composition of the prepared NR matrices in parts per hundred rubber (phr).

Sample ^1^	Vulcanization Time	NR	VA	SA	CBS	ZnO	6PPD
D1.2	5.1	100	1	0	0	0	0
D1.6	6	100	1.5	0	0	0	0
D2	6.2	100	2	0	0	0	0
S1.4	12.5	100	1	2	1.59	1.5	3
S1.7	12.6	100	1.5	2	2.38	1.5	3
S1.1	12.8	100	2	2	3.17	1.5	3

^1^ Sample name is given by the vulcanization agent used (D = DCP and S = Sulphur) and the crosslink density measured by swelling experiments in [10^−4^ cm^3^·mol^−1^].

## Data Availability

The original contributions presented in this study are included in the article. Further inquiries can be directed to the corresponding author.
